# The Treatment of Syphilis with Salvarsan

**Published:** 1912-03

**Authors:** 


**The Treatment of Syphilis with Salvarsan.** By Dr. WILHELM WECHSELMANN, with an Introduction by Dr. PAUL EHRLICH. Translated by ABR. L. WOLBARST, M.D. Pp. 175. London: Rebman Limited. 1911.

**The Experimental Chemotherapy of Spirilloses.** By PAUL EHRLICH and S. HATA. Translated by A. NEWBOLD, and revised by ROBERT W. FELKIN, M.D. Pp. xv, 181. London: Rebman Limited. 1911.

**"Salvarsan," or "606."** By W. HARRISON MARTINDALE, Ph.D., and W. WYNN WESTCOTT, M.B. Pp. xv, 77. London: H. K. Lewis. 1911.

These three books are all concerned with Ehrlich's researches into the curative properties of the organic arsenic compounds. The first book, by Wechselmann and Ehrlich, deals with the treatment of Syphilis only. It is well printed, and contains some excellent illustrations; but we are not carried away by the artistic merits of the latter. Syphilis under any treatment or none displays innumerable changes. Salvarsan or "606" is the subject of the book, and the account of its use and its dangers is given very fully. It is a book to master before venturing for the first time on the administration of Salvarsan in Syphilis. Experience will teach the administrator to temper its enthusiasm by his own observations.

In *Chemotherapy* a full description is given of many experiments with various arsenic compounds in syphilis, relapsing fever, spirillosis of fowls, and frambœsia. The greater part of the book is occupied by Hata's work on the effect of drugs on spirilla generally, and on syphilis in rabbits. There are short contributions by H. J. Nichols upon the effect of "606" on Spirochæta Pertenuis in the Animal Body, by Julius Iversen on the Chemotherapy of Relapsing Fever, by Bilter and Dreyer (of Cairo) on Relapsing Fever treated in the Cairo Infectious Hospital. The concluding remarks are by Ehrlich, in which he expresses the hope that by such methods as are laid down in this book "we may approach nearer and nearer to the principle of the *Therapia magna Sterilans.*"

Martindale and Westcott have collected into a handy and readable volume the essential parts of both the other books. English readers will probably prefer to depend upon this reliable summary.

